# Supplementary material for: Folic Acid and Vitamin B12 Prevent Deleterious Effects of Rotenone on Object Novelty Recognition Memory and Kynu Expression in an Animal Model of Parkinson’s Disease
Source: Genes (Basel). 2022 Dec 17;13(12):2397. doi: 10.3390/genes13122397 (PMC9778036; doi:10.3390/genes13122397)
Supplement: Supplementary file 1 [file genes-13-02397-s001.zip › genes-2018027-supplementary.pdf]

Article

Folic Acid and Vitamin B12 Prevent Deleterious Effects of Rotenone on Object Novelty Recognition Memory and *Kynu* Expression in an Animal Model of Parkinson's Disease.

Gabriela Canalli Kretzschmar [1,†], Adriano D. S. Targa [2,†], Sheila Coelho Soares-Lima [3], Priscila I. dos Santos [1], Lais S. Rodrigues [2], Daniel A. Macedo [2], Luis Felipe Ribeiro Pinto [3], Marcelo M. S. Lima [2] and Angelica Beate Winter Boldt [1,*]

[1] Laboratory of Human Molecular Genetics, Postgraduate Program in Genetics, Department of Genetics, Federal University of Paraná (UFPR), Centro Politécnico, Jardim das Américas, Curitiba 81531-990, PR, Brazil

[2] Laboratory of Neurophysiology, Department of Physiology, Federal University of Paraná (UFPR), Centro Politécnico, Jardim das Américas, Curitiba 81531-990, PR, Brazil

[3] Molecular Carcinogenesis Program, National Cancer Institute, Research Coordination, Rio de Janeiro 20231-050, RJ, Brazil

* Correspondence: angelicaboldt@gmail.com; Tel.: +55-(41)-3361-1553

† These authors contributed equally to this work.

Supplementary Table S1—Primers for *Kynu* Pyrosequencing.

Primer	5'- 3' Sequence	Nt	Tm ºC	%GC
Forward	AAGAGTTGGAAGAGGTTGTTAGT	23	60.8	39.1
Reverse -biotinylated	CTCCACCCTATAAAAAATTTACATATCAAC	30	59.1	30
Sequencing	GGTTGTTAGTAGAGTTAGAT	20	45.4	35

Sequence with marked primers

```
    AAGAGTTGGA AGAGGTTGTT AGT
    |||||||||| |||||||||| |||
                 GGTTGTT AGTAGAGTTA GAT
                 ||||||| |||||||||| |||
271 AAGAGTTGGA AGAGGTTGTT AGTAGAGTTA GATGTTTGGT AAGTTGGGGT GGGGATGGGG GAGGTGTYGT ATTTTGTTTA GTTTTTGTTG
                                                                        cg1
361 GTGGTTAGAA ATTTGTAATT YGATTTAATT GGGTGGGAGG AATTAAAGTT GATATGTAAA TTTTTTATAG GGTGGAG 438
                         cg2                                 ||| |||||||||| |||||||||| |||||||
                                                             CAA CTATACATTT AAAAAATATC CCACCTC
```

The primers were designed using the PyroMark Assay Design 2.0 (Qiagen), considering the promotor sequence of the *Kynu* gene in the RGSC 6.0/rn6 rat genome. The software provides a reliability score for the generated primers, and the highest option was chosen (93%). The analyzed CpG sites are represented in red. The *cg2* site is orthologous to *cg15836722* in the *KYNU* human promoter and is the closest CpG to the transcription start site.

Supplementary Table S2—Pyrosequencing PCR Protocol.

Reagents	[Initial]	[Final]
RNase-Free Water	0	0
PyroMark PCR Master Mix, 2x	2	1
CoralLoad Buffer, 10x	10	1
MgCl2 (mM)	25	2
Primer set (nmol)	2.54	0.2
DNA converted with bisulfite (ng/uL)	20	
**Total**		
**Expected products**		
Kynu	167 bp	

We used the PyroMark PCR kit (Qiagen) and evaluated the quality of PCR amplification in 1.5% agarose gel electrophoresis.

Supplementary Table S3—Primers for *Kynu* Expression Analysis.

Gene	Primer	5'-3'Sequence	Nt	Tm°C	%CG	bp
Kynu	F	TCTGTGACAAGCGAGAACCA	20	59.25	50	113
	R	TGTAGAGTCGAGTATGGCAGTAAG	24	59.42	45.83	
*Actb**	F	TGTCACCAACTGGGACGATA	20	58.37	50	166
	R	GGGGTGTTGAAAGGTCTCAAA	21	58.34	47.62	
*Hprt1**	F	GCAGACTTTGCTTTCCTTGG	20	57.57	50	81
	R	CGAGAGGTCCTTTTCACCAG	20	57.91	55	

* Primer sequences already described by Elfving et al., (2019).

Supplementary Table S4—qPCR Protocol.

Reagents	[Initial]	[Final]
Rnase-Free Water	0	0
GO Taq Master Mix, 2x	2	1
Primer Forward (uM)	10	10
Primer Reverse (uM)	10	10
cDNA (ng/uL)	12.5	12.5

Supplementary Figure S1—*Kynu* Methylation Levels of *cg1* and *cg2*.

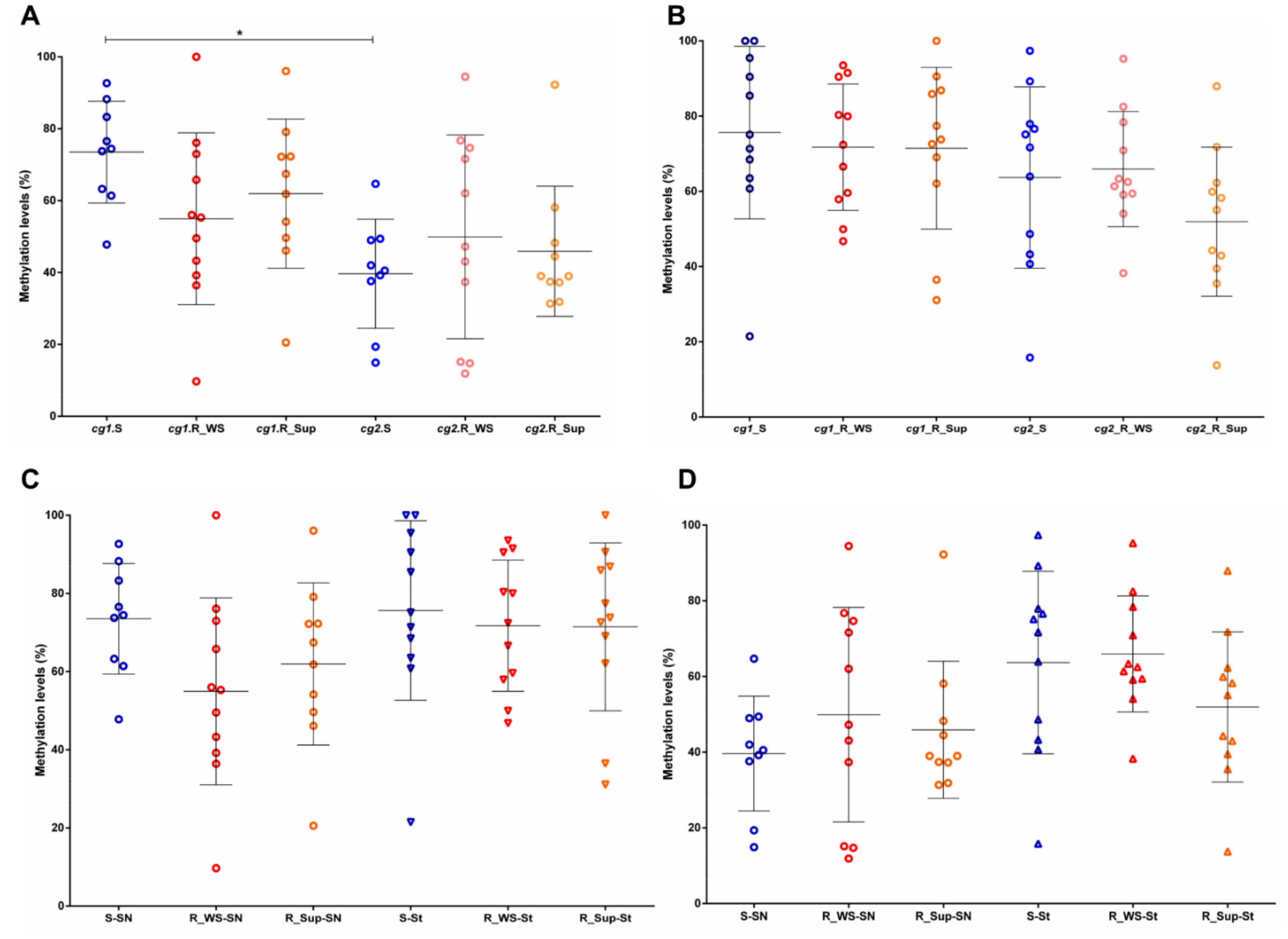

Figure S1. Methylation levels of *cg1* and *cg2* in the analyzed groups in (**A**) substantia nigra and (**B**) striatum; dark color = *cg1*; light color = *cg2*; (**C**) Methylation levels for *cg1* between analyzed groups and different brain regions; (**D**) Methylation levels for *cg2* between studied groups and different brain regions; S = sham; R_WS = rotenone without supplementation; R_Sup = rotenone supplemented. In B and C, the circles represent substantia nigra, and the triangles represent the striatum region. No significant differences were found between the groups in B, C, and D. * $p < .01$. Data are the mean with SD.

Supplementary Figure S2—Differences in Methylation Levels Between the Substantia Nigra and the Striatum.

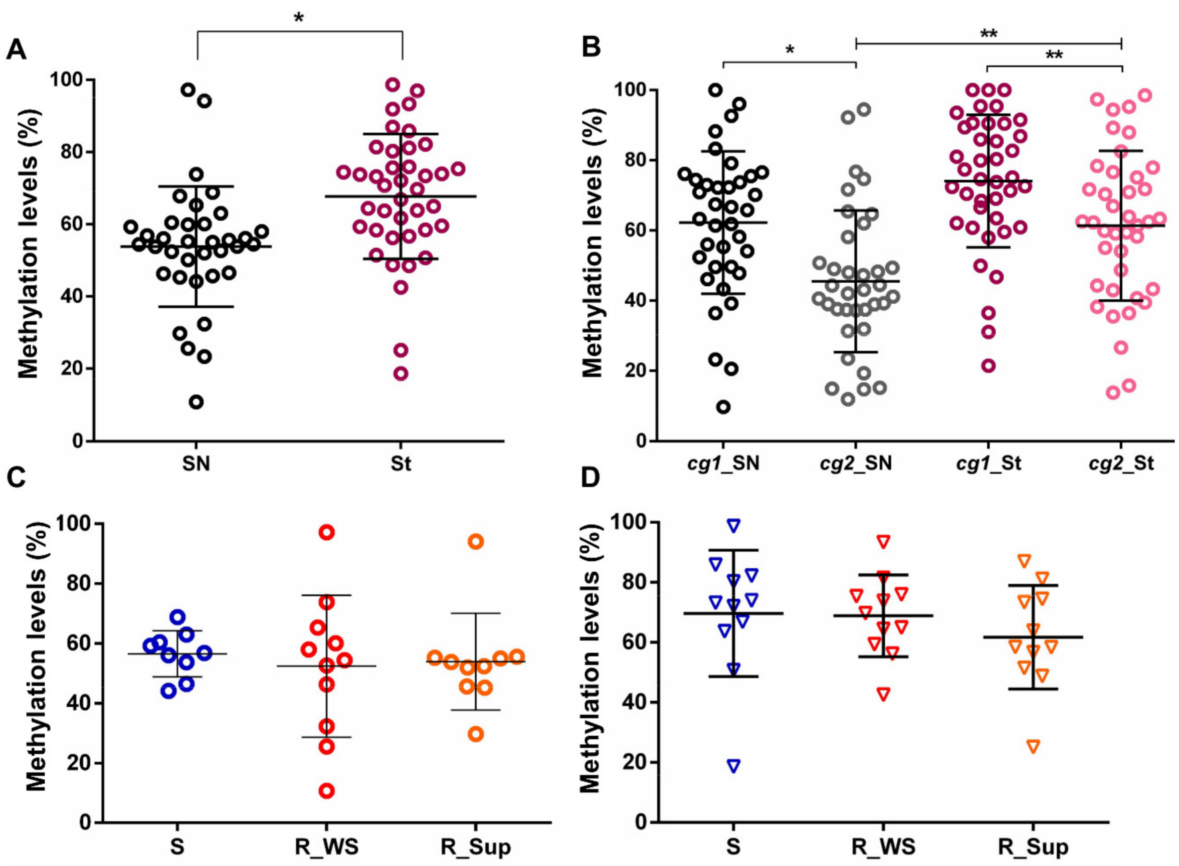

Figure S2. (**A**) Methylation mean (*cg1* and *cg2*) between the substantia nigra (SN) and the striatum (St). The methylation levels are higher in St (mean = 67.69 ± 17.31, n = 40) than SN (mean = 53.81 ± 16.66, n = 36). (**B**) Differences in methylation levels of two CpG sites analyzed between SN and St. *cg1* (SN: mean = 62.23 ± 20.34, n = 37; St: mean = 74.05 ± 18.86, n = 40) showed a higher methylation level than *cg2* (SN: mean = 45.51 ± 20.21, n = 36; St: = 61.32 ± 21.35, n = 40) in both brain regions. (**C**) Methylation mean (*cg1* and *cg2*) between the groups in SN. S = sham (mean = 56.58, 68.84 ± 44.20, n = 9); R_WS = rotenone without supplementation (mean = 52.44, 97.24 ± 10.82, n = 11); R_Sup = rotenone supplemented (mean = 53.93, 94.16 ± 29.79, n = 10). (**D**) Methylation mean (*cg1* and *cg2*) between the groups in St. S: mean = 69.67, 98.68 ± 18.66, n = 11; R_WS: = mean = 68.84, 93.38 ± 42.54, n = 11; R_Sup: mean = 61.70, 86.94 ± 25.14, n = 11. * $p < 0.01$; ** $p < 0.05$. Data are the mean with SD.

Supplementary Figure S3—Correlation Between *Kynu* Gene Expression and Methylation Levels.

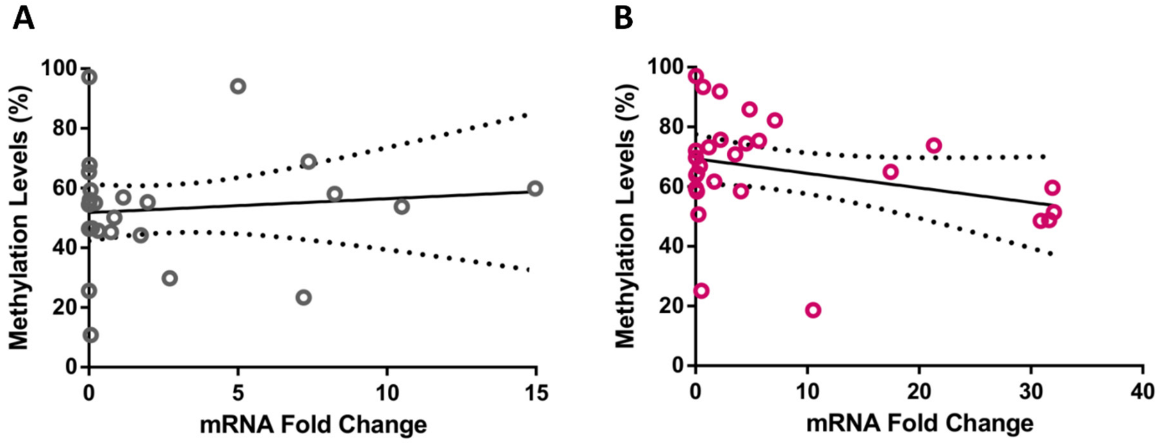

Figure S3. Absence of correlation between the expression of the *Kynu* gene and the CpG sites analyzed in (**A**) the substantia nigra (r = .09, p>.5, n = 25) and (**B**) the striatum (r = -0.31, p>.1, n = 28).

Supplementary Figure S4—*Kynu* Expression in the Substantia Nigra and the Striatum.

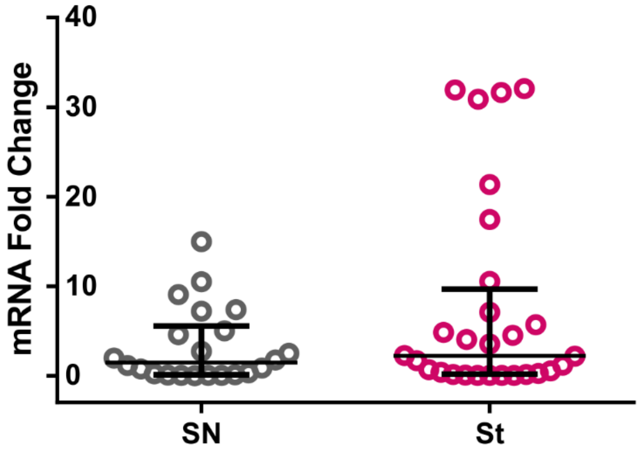

Figure S4. There were no significant changes in *Kynu* expression between the substantia nigra (median: 1.14, min: 0.004, max: 14.97, n = 22) and striatum (median: 2.21, min: 0.02, max: 32.06, n = 28). Data are the median with interquartile range. Min = minimum; Max = maximum.
